# COVID-19 mortality in Brazil, 2020-21: consequences of the pandemic inadequate management

**DOI:** 10.1186/s13690-022-01012-z

**Published:** 2022-12-19

**Authors:** Célia Landmann Szwarcwald, Cristiano Siqueira Boccolini, Wanessa da Silva de Almeida, Adauto Martins Soares Filho, Deborah Carvalho Malta

**Affiliations:** 1 Institute of Scientific and Technological Communication and Information in Health, Oswaldo Cruz Foundation, Rio de Janeiro, Brazil; 2 Faculty of Medicine, Federal University of Minas Gerais, Belo Horizonte, Minas Gerais Brazil; 3 School of Nursing, Federal University of Minas Gerais, Belo Horizonte, Minas Gerais Brazil

**Keywords:** COVID-19, Mortality, Maternal mortality, Number of years lost, Orphanhood, Epidemic denial, Vaccination

## Abstract

**Background:**

The COVID-19 pandemic brought countless challenges to public health and highlighted the Brazilian health system vulnerabilities in facing the emergency. In this article, we analyze data on COVID-19-related deaths in 2020-21 to show the epidemic consequences in Brazil.

**Methods:**

The Mortality Information System and the Live Birth Information System were the primary information sources. We used population estimates in 2020-21 to calculate COVID-19 specific mortality rates by age, sex, and educational level. Considering the total number of COVID-19 deaths in 2020-21, the COVID-19 proportional mortality (%) was estimated for each age group and sex. A graph of the daily number of deaths from January 2020 to December 2021 by sex was elaborated to show the temporal evolution of COVID-19 deaths in Brazil. In addition, four indicators related to COVID-19 mortality were estimated: infant mortality rate (IMR); maternal mortality ratio (MMR); number and rate of orphans due to mother’s COVID-19 death; the average number of years lost.

**Results:**

The overall COVID-19 mortality rate was 14.8 (/10,000). The mortality rates increase with age and show a decreasing gradient with higher schooling. The rate among illiterate people was 38.8/10,000, three times higher than a college education. Male mortality was 31% higher than female mortality. COVID-19 deaths represented 19.1% of all deaths, with the highest proportions in the age group of 40-59 years. The average number of years lost due to COVID-19 was 19 years. The MMR due to COVID-19 was 35.7 per 100,000 live births (LB), representing 37.4% of the overall MMR. Regarding the number of orphans due to COVID-19, we estimated that 40,830 children under 18 lost their mothers during the epidemic, with an orphans’ rate of 7.5/10,000 children aged 0-17 years. The IMR was 11.7 per 1000 LB, with 0.2 caused by COVID-19. The peak of COVID-19 deaths occurred in March 2021, reaching almost 4000 COVID-19 deaths per day, higher than the average number of deaths per day from all causes in 2019.

**Conclusions:**

The delay in adopting public health measures necessary to control the epidemic has exacerbated the spread of the disease, resulting in several avoidable deaths.

**Supplementary Information:**

The online version contains supplementary material available at 10.1186/s13690-022-01012-z.

## Background

From reporting the first SARS-CoV-2 infection in December 2019 to the end of April 2022, around 510 million infected and 6.2 million deaths from COVID-19 have been reported worldwide, with a lethality rate of 1.2% [1].

The COVID-19 pandemic evolution was directly related to the emergence of variants of concern (VOC) of the SARS-CoV-2 virus, classified as such due to the increasing transmissibility and worsened the epidemiological situation in places where they have expanded [2]. The variants Alpha (B.1.1.7), initially identified in the United Kingdom; Beta (B.1.351), discovered in South Africa; and P.1, which originated in the Brazilian state of Amazonas (Manaus), have been subjects of international concern [3]. All three variants have been associated with growing transmissibility. Lineage B.1.1.7 was associated with a higher lethality rate in the United Kingdom. The Manaus variant (P.1) was 1.7 to 2.4 times more transmissible than the other lineages of the virus [4–6]. The “Omicron” lineage became the predominant SARS-CoV-2 VOC in several countries from November 2021 and on, becoming the dominant variant circulating globally in 2022 [7].

The COVID-19 pandemic brought countless challenges to public health and highlighted the vulnerabilities of the health system and the weakness of the Brazilian public policies to face the emergency. Testing all suspected cases was one of the World Health Organization (WHO) recommendations for controlling the epidemic’s spread [8]. However, months after the arrival of the COVID-19 epidemic in Brazil, diagnostic testing supplies in public health services were still unavailable [9, 10]. The lack of free tests and the Brazilian Ministry of Health (MoH) management protocol of patients with mandatory testing only for severe acute respiratory cases caused the underestimation of COVID-19 cases and the spread of the disease. The fact that people with more severe symptoms presented higher chances of being tested compromised the analysis of the epidemic status and the monitoring of time-spatial trends [11].

Further, epidemic management was not in place at the national level, delegating social restrictions of physical contacts and other prevention measures to state and municipal governments [12, 13]. The activities implemented by the Federal Government focused on minimizing COVID-19 severity, setting a strategy of disseminating fake news, and using medicines that were proven ineffective against COVID-19 [14, 15]. Errant and contradictory messages from political agents of the Brazilian government formed a narrative that diverged and competed with guidelines and good practices based on evidence in health [16, 17].

Moreover, due to the difficulty to access official COVID-19 data on incident cases, hospitalizations, and deaths from the Ministry of Health, national press vehicles formed a consortium to provide transparency to COVID-19 data, starting to consolidate daily COVID-19 data received from the State Health Secretariats [18, 19].

An emblematic situation of the COVID-19 epidemic in Brazil took place in the city of Manaus, the capital of the state of Amazonas, located in the North Region. A group of researchers found that the virus evolved into the P.1 variant in mid-November 2020 [20]. With greater transmissibility, the Manaus variant spread rapidly nationwide and the likelihood of dying from COVID-19 increased shortly after the outbreak emergence of the P.1 variant. However, other factors also increased lethality, especially the health system unpreparedness to provide care for all cases in need of medical assistance [21]. In January 2021, Manaus experienced a severe health crisis, with a shortage of (mechanical) ventilators and hospital beds in Intensive Care Units (ICU), amid the dramatic lack of oxygen gas to treat people hospitalized with COVID-19 [22].

Among all these aspects, the delay in acquiring vaccines for epidemic control stands out [18, 23]. Although Brazil received proposals for the acquisition of vaccines in mid-2020 the Federal Government hesitated in negotiating these vaccines. The most decisive initiative for facing the COVID-19 epidemic and responsible for changing the trajectory of the epidemic in Brazil was the national vaccine production. The first vaccine (CoronaVac) was made available to healthcare professionals in January 2021 [24]. However, due to the country’s vaccine shortage, the Brazilian population’s vaccination followed slowly during the first half of 2021, scheduled by age group and prioritizing the elderly [25]. Vaccination was started among people less than 60 years only in May 2021.

The main problems in the management of the COVID-19 epidemic in Brazil are summarized in the Additional file 1: Appendix 1 of the supplementary material.

In April 2022, the Ministry of Health released official data from the 2020 Mortality Information System and the 2021 primary database. In this article, we analyze data on COVID-19-related deaths in Brazil in 2020-21 and use COVID-19 mortality indicators to show the impact and tragic consequences of the epidemic on the Brazilian population.

## Methods

Since January 2020, there has been a daily notification of all cases of COVID-19. The flow of sending COVID-19 data is similar to all other health information in Brazil. First, the municipal health secretaries consolidate COVID-19 data and send information to the state level. The state health secretaries consolidate the municipal information and send notified cases to the MoH.

In this study, the leading information sources were the Mortality Information System (SIM in Portuguese) and the Live Birth Information System (Sinasc in Portuguese). For population estimates, we used the projected population by the Brazilian Institute of Geography and Statistics (IBGE in Portuguese) for 2020-21, available at the site of the Department of Informatics of the Unified Health System (DATASUS), Ministry of Health [26]. The Population Projections for Brazil by sex and age are based on information on demographic dynamics derived from demographic censuses, household surveys, and administrative records of births and deaths.

Brazil adopted the WHO guidelines for COVID-19 in the national territory, preparing the Mortality Information System for implemented changes and publishing guidelines for the codification of COVID-19. There is a forum for coders to discuss the codification of causes of death in Brazil and focused on to improving the quality of the death certificate. Deaths due to COVID-19 were defined as having in any line of the medical certificate the ICD-10 code B34.2 accompanied by one of the two codes U07.1 (confirmed by laboratory testing) or U07.2 (diagnosed clinically or epidemiologically but laboratory testing is inconclusive or not available). Among cases for which the code B34.2 was not informed, but there is a record of U07.1, U07.2, or U92.1, the case record returned to the municipality for inconsistency correction [27].

We analyzed all deaths due to COVID-19 in Brazil reported to SIM in 2020 and 2021, according to the definition above and regardless of the classification of the underlying cause of death, such as maternal or HIV/AIDS death.

### Analysis of COVID-19 deaths and COVID-19 mortality rates

We used the IBGE population estimates in 2020-21 to calculate COVID-19-specific mortality rates by age and sex. The COVID-19-specific mortality rates were calculated by the ratio between the number of COVID-19 deaths and the number of inhabitants 2020 and 2021 combined for each age group (0-9, 10-19, 20- 29, 30-39, 40-49, 50-59, 60-69, 70-79, 80-89, 90+) and sex.

Mortality rates due to COVID-19 were also calculated by educational level among people aged 18 years or over (Illiterate; incomplete elementary school; complete elementary school; finished high school; finished college education). Deaths with missing data on education level were proportionally distributed in all categories. We used data from the National Health Survey, 2019 [28], to estimate the population distribution by educational level.

To verify the burden of COVID-19 mortality by age and sex, we considered the number of deaths for all causes in 2020 and 2021 and estimated the proportion (%) of COVID-19 deaths for each age group and sex.

A graph of the daily deaths from January 2020 to December 2021 by sex was elaborated to show the temporal evolution of COVID-19 deaths in Brazil.

### Indicators related to COVID-19 mortality

We estimated four indicators for the period from January 2020 to the end of December 2021:

i) The infant mortality rate due to COVID-19 (/1000 LB): The infant mortality rate was calculated as the average number of COVID-19 deaths in children under 1 year of age in 2020-2021 per 1000 live births (LB). The number of LB was informed to Sinasc, 2020 and adjusted by Sinasc underreporting [29].
ii) Maternal mortality ratio due to COVID-19 (/100,000 LB): Estimated by the ratio between the average number of maternal deaths from complications in pregnancy caused by COVID-19 in 2020-21 and the total number of live births in 2020, adjusted by Sinasc underreporting [29].
iii) The number of orphans (0-17 years old) resulting from the mother’s death by COVID-19: In this analysis, orphanhood was defined by the mother’s death. To estimate the number of children (< 18 years old) orphaned as a result of the mother’s death by COVID-19 in Brazil, we estimated mothers’ fertility rates at the same disaggregation level as COVID-19 deaths (5-year age groups) using data from the Sinasc in the years in which children younger than 18 years were born (2003–2020). Then, the number of COVID-19 deaths among females was multiplied by the estimated fertility rate for each 5-year age group. The number of orphans was calculated by the sum of the products between the number of female COVID-19 deaths and the fertility rates. Finally, we adjusted the number of orphans based on IBGE’s survival rate estimative to reach adulthood [30]. The orphans’ rate was calculated by the ratio between the estimated number of children under 18 years of age who lost their mother due to COVID-19 and the estimated population under 18 years old, 2020 [26]. The estimation method of the number of orphans associated with COVID-19 is presented in Additional file 1: Appendix 2 of supplementary material.
iv) The average number of years of life lost due to COVID-19: We used the complete 2020 IBGE mortality tables [30] as the source of information. The number of years lost by sex at each age was given by the life expectancy (from 0 to 80+) multiplied by the number of COVID-19 deaths at the same age. The average number of years of life lost was calculated by the ratio between the sum of the number of years lost and the total number of COVID-19 deaths. The estimation method of the average number of years of life lost by COVID-19 is presented in Additional file 1: Appendix 3 of supplementary material.

## Results

This study analyzed 631,697 deaths caused by COVID-19, 206,460 in 2020 and 425,237 in 2021. Table 1 shows the COVID-19 mortality rates by age and sex in 2020 and 2021 combined. The overall mortality rate from COVID-19 was 14.8 per 10,000 inhabitants. Mortality rates increase with age, reaching 83.5 and 161.5 per 10,000 inhabitants in the age groups of 70-79 years and 80 years or over, respectively, which means more than one death by COVID-19 for every 100 individuals aged 70 or over. COVID-19 mortality rates for males were higher than for females in all age groups over 20 years. Sex ratios show that overall COVID-19 male mortality was 31% higher than female mortality and 50% higher among people aged 40 years or over.

**Table 1:** COVID-19 Mortality Rates (/10,000 population) by sex and age group. Brazil, 2020-21

Age-group	COVID-19 Mortality Rates (/10,000)			Sex Ratio
	Males	Females	Total	
0-9	0.3	0.3	0.3	1.06
10-19	0.2	0.2	0.2	0.89
20-29	1.3	1.2	1.2	1.11
30-39	5.0	3.3	4.2	1.49
40-49	12.3	7.7	10.0	1.59
50-59	26.6	17.0	21.6	1.56
60-69	52.6	34.6	43.0	1.52
70-79	107.8	64.7	83.5	1.67
80-89	198.6	117.6	149.0	1.69
90+	287.9	176.9	212.3	1.63
Total	16.9	12.9	14.8	1.31

Information sources: 1 - Mortality Information System (SIM), Division of Health Surveillance, Ministry of Health. 2 - DATASUS. Projection of the population of Brazil by sex and age for the period 2000-2060. Brazil [26]

The COVID-19 mortality rates by educational level among people aged 18 years or over show a decreasing gradient with higher schooling, ranging from 38.8/10000 among people with no education to 13.0/10000 among people with a college education and decreases the higher the level of education. Among illiterate people, the rate was 38.8 per 10,000 inhabitants, three times higher than those with a college education (Table 2).

**Table 2:** COVID-19 Mortality Rates (/10,000 population) by educational level among people aged 18 years or over. Brazil, 2020-21

Educational Level	COVID-19 Mortality Rate (/10,000)
Illiterate	38.8
Incomplete elementary school	23.2
Complete elementary school	25.0
Finished high school	14.4
Finished college education	13.0
Total	19.8

Information sources: 1 - Mortality Information System (SIM), Division of Health Surveillance, Ministry of Health. 2 - National Health Survey, 2019

Regarding the proportional mortality (%) by age group and sex, the highest proportions were found among men and women aged 40-49 and 50-59. Deaths caused by COVID-19 in 2020-21 represent 19.1% of all deaths and correspond to 14.4% among the oldest (80 years or over) for both males and females. These proportions indicate that the age group of 40 to 59 years was more affected than the extreme categories (Table 3).

**Table 3:** COVID-19 Proportional Mortality (%) by sex and age group. Brazil, 2020-21

Age-group	COVID-19 Proportional Mortality (%)		
	Males	Females	Total
0-9	2.0	2.3	2.1
10-19	2.3	7.4	3.6
20-29	5.3	16.7	7.9
30-39	16.6	24.6	19.1
40-49	23.0	26.3	24.2
50-59	23.7	26.7	24.9
60-69	22.3	24.5	23.2
70-79	21.2	20.2	20.8
80-89	17.5	14.5	15.9
90+	12.5	9.8	10.8
Total	19.1	19.0	19.1

Information sources: 1 - Mortality Information System (SIM), Division of Health Surveillance, Ministry of Health

**Fig. 1:**
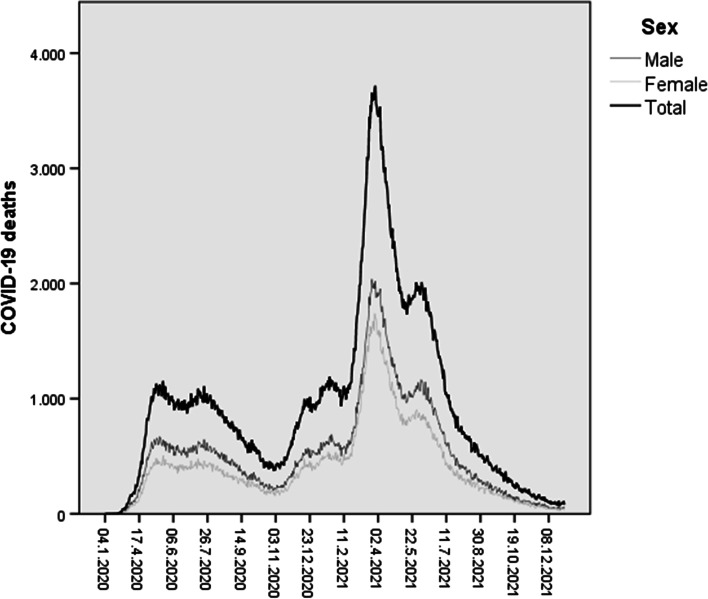
Time series of daily COVID-19 deaths by sex. Brazil, 2020-21

The daily time series of COVID-19 deaths in Brazil from January 2020 to December 2021 are shown in Fig. 1. The first COVID-19 wave in Brazil occurred in the first months of 2020 and reached more than 1000 daily COVID-19 deaths in May 2020. The first wave was followed by a slight decrease until November 2020, with the country’s onset of the second COVID-19 wave.

The epidemic peaked at the end of March 2021, reaching almost 4000 COVID-19 deaths per day and decreasing again to the level of 1000 COVID-19 deaths only in July of the same year. In the second half of 2021, a significant decrease in deaths was observed, reaching less than 100 daily deaths at the end of the year. The time trends by sex were the same as the total number of deaths. The number of deaths among men was always higher than among women, although nearing each other at the peak of COVID-19 deaths in Brazil.

As for the indicators related to COVID-19 mortality (Table 4), total infant mortality was 11.7 per 1000 live births (LB), with 0.2 per 1000 LB caused by COVID-19. The maternal mortality ratio (MMR) was much more affected. The overall estimate of the MMR was 95.4 per 100,000 LB, while the MMR due to COVID-19 was 35.7 per 100,000 LB, representing 37.4% of the total MMR. Considering the years lost among people who died from COVID-19, the average was 18.8 years overall, 18.1 for males and 19.8 for females. Regarding the number of orphans resulting from the mother’s death due to COVID-19, we estimated that 40,830 children under 18 lost their mothers during the epidemic in 2020-21, with an orphan rate of 7.5/10,000 children aged 0-17 years.

**Table 4:** Selected indicators related to COVID-19 mortality. Brazil, 2020-21

Indicator	Category	Estimate
Infant Mortality Rate (/1000 LB)	By COVID-19	0.2
	Due to other causes	11.5
	Total	11.7
Maternal Mortality Ratio (/100,000 LB)	By COVID-19	35.7
	Due to other causes	59.7
	Total	95.4
Average number of years lost	Males	18.1
	Females	19.8
	Total	18.8
Orphanhood due to mother’s death by COVID-19	Orphans’ rate (/10,000 children <18y)	7.5
	Number of orphans	40,830

*LB* live births

## Discussion

In this study, we considered COVID-19 deaths reported to the Mortality Information System, Brazil’s gold standard for registering deaths [31]. By the end of 2021, Brazil had recorded around 30 million cases and more than 630 thousand deaths by COVID-19. The “Sistema de Informação de Vigilância Epidemiológica” (SIVEP-influenza) is the epidemiological surveillance system for COVID-19 hospitalizations. Comparison between the two systems shows a higher number of deaths in the SIM than in SIVEP-influenza (2.7%) [32].

Brazil has 212 million inhabitants (3% of the global population) and reached almost 4000 COVID-19 deaths per day at the end of March 2021. This daily number of COVID-19 deaths was higher than the average number of deaths per day from all causes in 2019, around 3700 deaths per day. The spread of the epidemic in late 2020 occurred after the Manaus variant (P.1) introduction with higher transmissibility [20]. The continuity of air travels between Manaus and the rest of Brazil, crowding events at the end of the year, such as Christmas and New Year’s Eve parties, and school return around February/March after summer vacations are elements that favor the transmission of the virus. Mainly, the lack of national coordination in implementing social distancing measures has contributed to the rapid spread of cases. In turn, inadequate management of the COVID-19 epidemic caused an unprecedented crisis in Brazilian health. The unavailability of equipment, inpatient beds, places in ICUs, and health care teams to meet emergency health needs has undoubtedly aggravated the COVID-19 lethality in the country [33–35].

In this study, the results stratified by educational level showed higher COVID-19 mortality rates among less educated people. A possible explanatory hypothesis is that individuals with lower academic grades, generally working outside the home and unable to stop working during the epidemic, became more exposed to COVID-19 infection [11]. Additionally, the urban population of low socioeconomic status is concentrated in slum communities in the large Brazilian metropolises, preventing compliance with social distancing measures and isolation of cases diagnosed with COVID-19 [36]. Differences in mortality rates by educational level, with higher burden among lower educational level individuals, reflect the uneven impact of the epidemic on Brazilian socially disadvantaged families [37]. As has been discussed, the lower education and income population have less access to health services and a more significant number of comorbidities, increasing vulnerability to COVID-19 [38].

This study results emphasized the epidemic impact on mortality among people aged 40-59. The highest proportions of COVID-19 deaths were found in this age group, and the average number of years lost due to COVID-19 was approximately 19 years of life. One of the explanatory factors for the more significant burden of deaths in the non-elderly Brazilian population was the delayed vaccination of this population group. The peak of COVID-19 deaths at the end of March 2021 occurred in high SARS-CoV-2 infection transmissibility when the population under 60 years of age had not yet been vaccinated [25].

From a social viewpoint, the distribution of infection exposure is unequal by socioeconomic level, regarding not only working outside the home and using public transportation but also prevention behaviors [39]. In Brazil, most individuals in the working-age range continued to perform work outside the home [40], resulting in greater exposure to the virus and a disproportionate increase in deaths in the economically active population.

A study carried out in the state of Amazonas analyzed the mortality profile before and after the emergence of the P.1 variant in the Amazonas state. Like our findings, the comparison of the two epidemiological periods, April/May 2020 and January 2021, when the new variant started to predominate, showed a higher incidence of COVID-19 cases in the younger age groups. An increase was also found in the proportion of women in severe acute respiratory infection cases. The proportion of COVID-19 deaths among people aged 20-59 years increased for both males and females, and the lethality rate among those aged 20-39 years during the second wave was 2.7 times higher than the rate observed in the first wave [20]. Between March 2020 and April 2022, Brazil had three waves of COVID-19 (considering a combination of cases and deaths per million inhabitants), remaining at high levels until cooling down in December 2021.

Another aspect of young women’s mortality was increased maternal deaths due to pregnancy complications caused by COVID-19. Our study evidenced an over-mortality from COVID-19 of 37% on maternal mortality from other causes. The MMR in Brazil, which was already high – 62.1/100,000 LB in 2019 [41], reached a value close to 1 per 1000 live births after adding COVID-19 maternal deaths. It is necessary to mention that the number of miscarriages in pregnant women with COVID-19 has not been analyzed and would significantly increase the burden of COVID-19 on births and maternal health.

Of the total number of infant deaths (62,314) in the years 2020 and 2021, 958 were caused by COVID-19 (1.5%), with little impact on the infant mortality rate. Possible explanatory hypotheses are an increase in stillbirths and miscarriages before the child is born, besides the growth in maternal deaths during pregnancy. Also, there was a decrease in fertility as many women of childbearing age avoided pregnancy in these years. Further infant mortality studies are being conducted to better understand the small effect on the infant mortality rate.

The average number of lives lost by COVID-19 achieved almost 19 years overall and was higher in younger people since life expectancy decreases with age. As to the gender difference, the average number of years lost is also more significant because the life expectancy is higher among females.

In this study, we estimated that 7.5 out of every 10,000 children under 18 lost their mothers during the COVID-19 epidemic, totaling about 40,830 orphans due to COVID-19. Our result is similar to that found in the study by Unwin et al. [42], who compared COVID-19 orphans’ estimates in several countries. The findings showed that the orphans’ rate in Brazil was the 6th highest among the countries considered in the study. Furthermore, the number of orphans by one or both parents has been estimated in Brazil, between March 2020 and October 2021, in approximately 170,000 children under 18 years of age [42]. Admittedly, the death of a parent, particularly the mother, is linked to adverse outcomes throughout life and has severe consequences for the family’s well-being, profoundly affecting the family structure and dynamics [43]. The experience of the HIV/AIDS epidemic has shown that orphaned children are particularly vulnerable in terms of emotional and behavioral problems, requiring intervention programs to improve the psychological consequences of losing a parent [44].

The COVID-19 pandemic has had negative impacts globally, some with more dramatic consequences than others. Among the lessons learned, disease denial has undoubtedly influenced the performance of national health systems in mitigating the harmful impact of the COVID-19 pandemic. In Brazil [45], as in other countries [46–48], the lack of information on incident cases and deaths by COVID-19 and the delay in adopting the public health measures necessary to control the epidemic has exacerbated the spread of the disease, resulting in avoidable losses of human lives. Denial in some countries leads critically ill patients with severe health risks to a state of abandonment, with no possibility of receiving medical care [47].

From an individual perspective, skepticism towards COVID-19 can be conceptualized as the denial of the disease’s severity and the perception that the pandemic is exaggerated or a hoax. In a study in the United States, skepticism was strongly associated with a reduction in prevention behaviors [49]. Individual freedom to adopt prevention behaviors has been widely discussed in the face of a highly transmissible infection since the personal option can harm others or society in general [50].

A limitation of this study is that we used preliminary mortality data from 2021, which will still undergo a process of improving the quality of the cause of death. Possibly with less impact on COVID-19 mortality as a surveillance alert process for timely notification of deaths by COVID-19 has been implemented in Brazil since May 2020 [51].

At the beginning of 2021, Brazil woke up to the urgent need to vaccinate the population as a strategy to control the epidemic. The national production of large-scale vaccines for COVID-19 from imported active pharmaceutical ingredients (API) began in the first months of 2021. The acquisition of imported vaccines, alongside the gradual increase in national production and the distribution of the vaccine in the primary health care (PHC) units of the Unified Health System (SUS), have contributed enormously to the mass vaccination of the Brazilian population. As of March 2022, the percentage of people with one dose of vaccine is 83.2%, the rate of complete immunization (2 doses) is 76.9, and 42.0% have a booster dose. Additionally, the vaccine technology was internalized, and the first doses of the fully nationalized vaccine were delivered in February 2022.

After the COVID-19 toll on the Brazilian population and the subsequent sadness and grief over the loss of family and friends, the worsening of socioeconomic conditions, the stressful situations, and the distance from loved ones, it is time to rebuild. It takes courage to overcome new challenges, smile again, enjoy the freedom to come and go, embrace others, and value life.

## Conclusions

Since the beginning of the epidemic, Brazil has reported around 30 million cases and more than 630 thousand deaths by COVID-19 [25]. The disease denial has undoubtedly influenced the performance of the national health system in mitigating the harmful impact of the COVID-19 epidemic in Brazil, especially the unpreparedness to provide care for all cases in need of medical assistance. The subsequent delay in adopting the public health measures necessary to control the epidemic has exacerbated the spread of the disease, resulting in several avoidable deaths.

## Supplementary Information

**Additional file 1.**

## Data Availability

Not applicable.

## Abbreviations

COVID-19: Coronavirus Disease 2019
SARS-CoV-2: Severe Acute Respiratory Syndrome Coronavirus 2
VOC: Variants of Concern
MoH: Brazilian Ministry of Health
ICU: Intensive Care Units
SIM: Brazilian Mortality Information System
Sinasc: Brazilian Live Birth Information System
IBGE: Brazilian Institute of Geography and Statistics
DATASUS: Department of Informatics of the Brazilian Unified Health System
WHO: World Health Organization
ICD-10: International Statistical Classification of Diseases and Related Health Problems 10th Revision
HIV: Human Immunodeficiency Virus
AIDS: Acquired Immunodeficiency Syndrome
LB: Live Births
IMR: Infant Mortality Rate
MMR: Maternal Mortality Ratio
API: Active Pharmaceutical Ingredients
PHC: Primary Health Care
SUS: Brazilian Unified Health System
