# Supplementary material for: COVID-19 mortality in Brazil, 2020-21: consequences of the pandemic inadequate management
Source: Arch Public Health. 2022 Dec 19;80:255. doi: 10.1186/s13690-022-01012-z (PMC9762984; doi:10.1186/s13690-022-01012-z)
Supplement: Supplementary file 1 — Additional file 1. [file 13690_2022_1012_MOESM1_ESM.docx]

**Supplementary Material**

**Appendix 1**

In this section, we summarize the main problems in the management of the COVID-19 epidemic in Brazil.

**1. Low COVID-19 testing**

On February 26, 2020, the first case of New Coronavirus was confirmed in São Paulo, Brazil, with a history of travel to the Lombardy region of Italy. Subsequent genomic analysis carried out on representative samples from all over Brazil detected more than 100 points of introduction of SARS-CoV-2 from other countries. People coming from Europe spread the disease throughout the national territory between February 22 and March 11, 2020, evidencing the lack of surveillance in ports and airports until that moment.

Months after the arrival of the COVID-19 epidemic in Brazil, diagnostic testing supplies in public health services were still unavailable in public primary health units (Prado et al., 2020; Reis et al., 2020). With the shortage of diagnostic tests in the public sector throughout 2020, it was not possible to implement strategies for tracking cases or isolating those infected. Additionally, the Brazilian Ministry of Health (MoH) management protocol of patients requiring diagnosis tests only for symptomatic cases accelerated the spread of the disease (Almeida et al., 2021).

The solution was the adoption of a passive surveillance based on the confirmed cases, resulting in a great limitation of strategies to identify risk factors for exposure and infection.

**2. Use of a hospital-centered approach to face the epidemic**

The national response to the epidemic predominantly centered on the biomedical and hospital-centric approach. At the beginning of the COVID-19, there was no Personal Protective Equipments (PPE) for health professionals and the primary care units lacked diagnosis tests and oximeters. The potential of Primary Health Care (PHC) in coping with the epidemic was underutilized in favor of greater targeting and investment in Intensive Care Units, hospital beds and field hospitals. Such a choice deprived the country of using a network of health services spread throughout the country, which would have immense potential to support surveillance actions such as testing, contact tracing and isolation of infected people (ABRASCO, 2022).

**3. Epidemic denial by the Federal Government**

The disease denial has undoubtedly influenced the performance of the Federal Government in mitigating the harmful impact of the COVID-19 epidemic. The President of Brazil, in office since the beginning of the epidemic, minimized the seriousness of the epidemic and defended the maintenance of economic activities, claiming that the spread of the virus would lead to the development of group immunity. However, the “natural” spread control has never happened. Besides, health ministers were changed when they opposed the President’s positions. In this scenario, both the use of masks and the adoption of measures to restrict physical contact were discouraged by the Federal Government. The implemented activities focused on minimizing COVID-19 severity and setting a strategy of disseminating fake news (Lancet, 2020).

**4. Promotion of ineffective treatments**

Among the most controversial topics, the indication of drugs for the early treatment of the disease stood out, especially chloroquine and hydroxychloroquine. This kind of treatment, advocated and sponsored by the Federal Government itself, was rejected by the scientific community due to the lack of evidence on its effectiveness in combating the virus (Elavarasi et al., 2020). However, a technical note on the Ministry of Health legitimized the indiscriminate and irrational use of these drugs, despite not bringing benefits to the control of the epidemic in the country (ABRASCO, 2022).

**5. Absence of national coordination in implementing protection measures**

The COVID-19 impact in Brazil was aggravated by the absence of a technically and politically committed national coordination. The lack of a unified communication policy resulted in contradictory messages, which diverged and competed with guidelines and good practices based on evidence in health (Campos, 2020; Lancet, 2020). In the absence of federal coordination of actions to control the epidemic spread, it was up to governors and mayors to implement control measures, including social distancing and the mandatory use of masks. The administrative autonomy of states and municipalities in areas such as health, education and commerce is provided for in the Federal Constitution and restricted the possibility of direct interference by the Federal Government in local government decisions (ABRASCO, 2022).

The actions adopted by the municipalities included measures to restrict physical contact between people, more or less strict depending on the epidemic status, and the mandatory use of masks. In several cities, non-essential services, commercial establishments and schools were closed for a long period. Municipalities and communities have also built successful alternatives to face the epidemic, such as the experience of the National Council of Health Secretaries (CONASS) and the Northeast Consortium, which minimized the dramatic consequences of the epidemic. Many positive experiences developed by civil society emerged as alternatives to the Government failure to adequately control the disease (ABRASCO, 2022).

**6. Inadequate management of the health system**

Other factors have also exacerbated the spread of the disease and the number of deaths, especially the health system unpreparedness to provide care for all cases in need of medical assistance (Souza et al., 2020). In January 2021, Manaus experienced a severe health crisis, with a shortage of (mechanical) ventilators and hospital beds in Intensive Care Units (ICU), amid the dramatic lack of oxygen gas to treat people hospitalized with COVID-19 (Buss et al., 2021). In the emergency crisis, family members of patients hospitalized with COVID-19 refilled themselves empty oxygen tanks in private companies (ABRASCO, 2022).

The unavailability of equipment, inpatient beds, places in ICUs, and health care teams to meet emergency health needs has undoubtedly contributed to increase the number of COVID-19 deaths in the country (Silva et al., 2021; Boschiero et al., 2021; Brizzi et al., 2022).

**7. Lack of information of incident cases and death**

Since the beginning of the COVID-19 epidemic in Brazil, COVID-19 mortality data, hospitalizations and information on incident COVID-19 cases from the Ministry of Health were not officially available (Prado et al., 2020; Reis et al., 2020). Additionally, contrary to the global rule, the Brazilian MoH changed the criterion for counting deaths from COVID-19 in June 2020. This was the highest point of distrust with the MoH information. In June 2020, national press vehicles formed a consortium to provide transparency to COVID-19 data, starting to consolidate daily COVID-19 data received from the State Health Secretariats (Xavier et al., 2022; Taylor, 2022).

**8. Delay of vaccination against COVID-19**

Brazil had the opportunity to acquire vaccines in August 2020, but the Federal Government did not believe in vaccination as a way to control the COVID-19 epidemic. At the beginning of 2021, the Federal Government woke up to the need to vaccinate the population as a strategy to control the epidemic. The most decisive initiative for facing the COVID-19 epidemic and responsible for changing the trajectory of the epidemic in Brazil was the vaccine production carried out by Butantan Institute and Oswaldo Cruz Foundation (Fiocruz). While Butantan Institute opted for a strategy of immediate acquisition of a solution that would allow for faster provision of the vaccine, having the merit of having been the first vaccine (Coronavac) administered in the country, Fiocruz sought a sustainable solution that would allow technological appropriation and consequent independence in the production of the biopharmaceutical. The combination of the two strategies (Butantan and Fiocruz) allowed for faster access to immunization against COVID-19, followed by a sustained production of doses during the epidemic. In addition, the vaccine distribution in the primary health care (PHC) units has contributed enormously to the mass vaccination of the Brazilian population (ABRASCO, 2022).

**References**

Almeida WDS, Szwarcwald CL, Malta DC, Barros MBA, Souza Júnior PRB, Azevedo LO, et al. Changes in Brazilians’ socioeconomic and health conditions during the COVID-19 pandemic. Rev Bras Epidemiol. 2021;23:e200105.

Associação Brasileira de Saúde Coletiva – ABRASCO. Dossiê ABRASCO. Pandemia de COVID-19. 2022. https://materiais.abrasco.org.br/publicacoes-abrascao/. Access 06 Dec 2022.

Boschiero MN, Palamim CVC, Ortega MM, Mauch RM, Marson FAL. One year of coronavirus disease 2019 (COVID-19) in Brazil: A political and social overview. Ann Glob Health. 2021;87(1):44.

Brizzi A, Whittaker C, Servo LMS, Hawryluk I, Prete CA Jr, de Souza WM, et al. Spatial and temporal fluctuations in COVID-19 fatality rates in Brazilian hospitals. Nat Med. 2022;28:1476–85.

Buss LF, Prete CA Jr, Abrahim CMM, Mendrone A Jr, Salomon T, de Almeida-Neto C, et al. Three-quarters attack rate of SARS-CoV-2 in the Brazilian Amazon during a largely unmitigated epidemic. Science. 2021;371(6526):288–92.

Campos GWS. O pesadelo macabro da Covid-19 no Brasil: entre negacionismos e desvarios. Trab Educ Saúde. 2020;18(3):e00279111.

Elavarasi A, Prasad M, Seth T, Sahoo RK, Madan K, Nischal N, et al. Chloroquine and Hydroxychloroquine for the Treatment of COVID-19: a Systematic Review and Meta-analysis. J Gen Intern Med. 2020;35(11):3308–14.

Prado MFD, Antunes BBP, Bastos LDSL, Peres IT, Silva AABD, Dantas LF, et al. Analysis of COVID-19 under-reporting in Brazil. Rev Bras Ter Intensiva. 2020;32(2):224–8.

Reis RF, de Melo QB, de Oliveira CJ, Gomes JM, Rocha BM, Lobosco M, et al. Characterization of the COVID-19 pandemic and the impact of uncertainties, mitigation strategies, and underreporting of cases in South Korea, Italy, and Brazil. Chaos Solitons Fractals. 2020;136:109888.

Silva LL, de Carvalho DA, de Andrade L, Iora PH, Rodrigues Ramajo GL, Peres Gualda IA, et al. Emergency care gap in Brazil: geographical accessibility as a proxy of response capacity to tackle COVID-19. Front Public Health. 2021;9:740284.

Souza PMM, Gerson G, Soares CEL, Souza SG, Dias JS, Melo DN, et al. COVID-19 home deaths without medical assistance in northeastern Brazil. Am J Trop Med Hyg. 2020;104(2):514–8.

Taylor L. Covid-19: Brazil sees omicron cases soar but data blackout obscures true impact. BMJ. 2022;376:o133.

The Lancet. COVID-19 in Brazil: “so what?”. Lancet. 2020;395(10235):1461.

Xavier DR, Lima E, Silva E, Lara FA, E Silva GRR, Oliveira MF, et al. Involvement of political and socio-economic factors in the spatial and temporal dynamics of COVID-19 outcomes in Brazil: A population-based study. Lancet Reg Health Am. 2022;10:100221.

**Appendix 2**

**Estimation of maternal orphanhood due to COVID-19**

To estimate the number of children (<18 years old) orphaned as a result of the mother’s death by COVID-19 in Brazil, we estimated mothers’ fertility rates at the same disaggregation level as COVID-19 deaths (5-year age groups) in the years in which children younger than 18 years were born (2003-2020).

In Brazil, the number of live births by women of different ages is available annually in the Live Births Information System (Sinasc). In the first step, fertility rates were estimated from 2003 to 2020 by the mother's age group using data from the System of Live Birth Information (Sinasc). We assumed that fertility in 2021 was the same as in 2020 (Table A2).

To calculate the maternal orphanhood associated to COVID-19, the number of COVID-19 deaths among females was multiplied by the estimated fertility rates for each 5-year age-group. The total number of orphans was calculated by the sum of the products between the number of COVID-19 deaths and fertility rates (Table A1). Finally, we adjusted the number of orphans based on IBGE’s estimates of survival rate (0.98) to reach adulthood (IBGE, 2018). The number of living children under the age of 18 who were orphaned by the death of their mothers from COVID-19 was approximately 40,830.

The orphans` rate (7.5/10000) was calculated by the ratio between the estimated number of children under 18 years of age who lost their mother due to COVID-19 (40,830) and the estimated population under 18 years old (DATASUS, 2022).

**Table A2. Estimation of the number of orphans resulting from mother death by COVID-19**

| **Age group** | **Fertility Rate** | **Number of COVID-19 deaths among females** | **Number of orphans associated to COVID-19*** |
| --- | --- | --- | --- |
| 15-19 | 0.16 | 560 | 89 |
| 20-24 | 0.55 | 1377 | 761 |
| 25-29 | 1.00 | 2579 | 2577 |
| 30-34 | 1.45 | 4355 | 6316 |
| 35-39 | 1.41 | 7168 | 10087 |
| 40-44 | 0.97 | 10168 | 9850 |
| 45-49 | 0.53 | 13182 | 6965 |
| 50-54 | 0.21 | 17514 | 3751 |
| 55-59 | 0.05 | 24615 | 1271 |
| Total | | | 41666 |

* Number of children borne in the period 2003-2020 by women who died from COVID-19

**References**

Instituto Brasileiro de Geografia e Estatística (IBGE). Projeção da População das Unidades da Federação por sexo e grupos de idade: 2010-2060. 2018. https://www.ibge.gov.br/estatisticas/sociais/populacao/9109-projecao-da-populacao.html?edicao=21830&t=resultados. Accessed 2 Dec 2022.

Departamento de Informática do SUS - DATASUS. Ministério da Saúde/SVS/DASNT/CGIAE. Informações de saúde. População residente - Estudo de estimativas populacionais por município, idade e sexo 2000-2021. Brasil; 2022. http://tabnet.datasus.gov.br/cgi/deftohtm.exe?ibge/cnv/popsvsbr. Accessed 2 May 2022.

**Appendix 3**

**Estimation of the average number of years lost due to COVID-19 deaths in the period 2020-2021 by sex in Brazil**

The number of years lost by sex at each age was given by the life expectancy at that age (from 0 to 85+) multiplied by the number of COVID-19 deaths at the same age. The average number of years of life lost was calculated by the ratio between the sum of the number of years lost and the total number of COVID-19 deaths. The estimation method is presented in Table A3.

**Table A3: Estimation of the average number of years lost due to COVID-19 deaths. Brazil, 2020-21**

| **Age (x)** | **Males** | | | **Females** | | |
| --- | --- | --- | --- | --- | --- | --- |
|  | **Number of COVID-19 deaths** | **Life Expectancy at age x** | **Number of years lost** | **Number of COVID-19 deaths** | **Life Expectancy at age x** | **Number of years lost** |
| 0 | 524 | 73.3 | 38409 | 432 | 80.3 | 34690 |
| 1 | 91 | 73.2 | 6661 | 98 | 80.2 | 7860 |
| 2 | 37 | 72.3 | 2675 | 51 | 79.2 | 4039 |
| 3 | 35 | 71.3 | 2496 | 31 | 78.3 | 2427 |
| 4 | 29 | 70.4 | 2042 | 37 | 77.3 | 2860 |
| 5 | 37 | 69.4 | 2568 | 29 | 76.3 | 2213 |
| 6 | 33 | 68.4 | 2257 | 19 | 75.3 | 1431 |
| 7 | 20 | 67.4 | 1348 | 25 | 74.3 | 1858 |
| 8 | 24 | 66.5 | 1596 | 23 | 73.4 | 1688 |
| 9 | 25 | 65.5 | 1638 | 24 | 72.4 | 1738 |
| 10 | 22 | 64.5 | 1419 | 20 | 71.4 | 1428 |
| 11 | 24 | 63.5 | 1524 | 29 | 70.4 | 2042 |
| 12 | 26 | 62.5 | 1625 | 32 | 69.4 | 2221 |
| 13 | 61 | 61.5 | 3752 | 39 | 68.4 | 2668 |
| 14 | 43 | 60.6 | 2606 | 52 | 67.4 | 3505 |
| 15 | 69 | 59.6 | 4112 | 79 | 66.5 | 5254 |
| 16 | 82 | 58.6 | 4805 | 97 | 65.5 | 6354 |
| 17 | 100 | 57.7 | 5770 | 103 | 64.5 | 6644 |
| 18 | 118 | 56.8 | 6702 | 116 | 63.5 | 7366 |
| 19 | 133 | 55.9 | 7435 | 165 | 62.6 | 10329 |
| 20 | 180 | 55.0 | 9900 | 203 | 61.6 | 12505 |
| 21 | 223 | 54.1 | 12064 | 268 | 60.6 | 16241 |
| 22 | 243 | 53.2 | 12928 | 253 | 59.6 | 15079 |
| 23 | 303 | 52.4 | 15877 | 301 | 58.7 | 17669 |
| 24 | 384 | 51.5 | 19776 | 352 | 57.7 | 20310 |
| 25 | 474 | 50.6 | 23984 | 429 | 56.7 | 24324 |
| 26 | 559 | 49.7 | 27782 | 449 | 55.8 | 25054 |
| 27 | 575 | 48.8 | 28060 | 516 | 54.8 | 28277 |
| 28 | 712 | 47.9 | 34105 | 561 | 53.8 | 30182 |
| 29 | 806 | 47.0 | 37882 | 624 | 52.9 | 33010 |
| 30 | 925 | 46.1 | 42643 | 708 | 51.9 | 36745 |
| 31 | 1048 | 45.2 | 47370 | 809 | 50.9 | 41178 |
| 32 | 1224 | 44.4 | 54346 | 863 | 50.0 | 43150 |
| 33 | 1397 | 43.5 | 60770 | 950 | 49.0 | 46550 |
| 34 | 1556 | 42.6 | 66286 | 1025 | 48.1 | 49303 |
| 35 | 1750 | 41.7 | 72975 | 1231 | 47.1 | 57980 |
| 36 | 1981 | 40.8 | 80825 | 1231 | 46.1 | 56749 |
| 37 | 2162 | 39.9 | 86264 | 1378 | 45.2 | 62286 |
| 38 | 2455 | 39.0 | 95745 | 1540 | 44.2 | 68068 |
| 39 | 2618 | 38.1 | 99746 | 1788 | 43.3 | 77420 |
| 40 | 2690 | 37.2 | 100068 | 1741 | 42.4 | 73818 |
| 41 | 2976 | 36.3 | 108029 | 1953 | 41.4 | 80854 |
| 42 | 3173 | 35.5 | 112642 | 2022 | 40.5 | 81891 |
| 43 | 3317 | 34.6 | 114768 | 2124 | 39.5 | 83898 |
| 44 | 3509 | 33.7 | 118253 | 2328 | 38.6 | 89861 |
| 45 | 3808 | 32.9 | 125283 | 2417 | 37.7 | 91121 |
| 46 | 3872 | 32.0 | 123904 | 2440 | 36.8 | 89792 |
| 47 | 3916 | 31.2 | 122179 | 2590 | 35.9 | 92981 |
| 48 | 4349 | 30.3 | 131775 | 2800 | 35.0 | 98000 |
| 49 | 4522 | 29.5 | 133399 | 2935 | 34.1 | 100084 |
| 50 | 4726 | 28.7 | 135636 | 3027 | 33.2 | 100496 |
| 51 | 5047 | 27.8 | 140307 | 3256 | 32.3 | 105169 |
| 52 | 5407 | 27.0 | 145989 | 3609 | 31.4 | 113323 |
| 53 | 5558 | 26.2 | 145620 | 3705 | 30.5 | 113003 |
| 54 | 5754 | 25.4 | 146152 | 3917 | 29.7 | 116335 |
| 55 | 6419 | 24.6 | 157907 | 4498 | 28.8 | 129542 |
| 56 | 6708 | 23.9 | 160321 | 4563 | 27.9 | 127308 |
| 57 | 6982 | 23.1 | 161284 | 5019 | 27.1 | 136015 |
| 58 | 7329 | 22.3 | 163437 | 5114 | 26.2 | 133987 |
| 59 | 7478 | 21.6 | 161525 | 5421 | 25.4 | 137693 |
| 60 | 7229 | 20.8 | 150363 | 5408 | 24.6 | 133037 |
| 61 | 7304 | 20.1 | 146810 | 5395 | 23.8 | 128401 |
| 62 | 7749 | 19.4 | 150331 | 5954 | 22.9 | 136347 |
| 63 | 8041 | 18.7 | 150367 | 6115 | 22.1 | 135142 |
| 64 | 8284 | 18.0 | 149112 | 6181 | 21.3 | 131655 |
| 65 | 8362 | 17.3 | 144663 | 6392 | 20.6 | 131675 |
| 66 | 8916 | 16.6 | 148006 | 6806 | 19.8 | 134759 |
| 67 | 8832 | 15.9 | 140429 | 6714 | 19.0 | 127566 |
| 68 | 8966 | 15.3 | 137180 | 6787 | 18.3 | 124202 |
| 69 | 8809 | 14.6 | 128611 | 6582 | 17.5 | 115185 |
| 70 | 9133 | 14.0 | 127862 | 6870 | 16.8 | 115416 |
| 71 | 8895 | 13.4 | 119193 | 6617 | 16.1 | 106534 |
| 72 | 8857 | 12.8 | 113370 | 6722 | 15.4 | 103519 |
| 73 | 8728 | 12.2 | 106482 | 6890 | 14.8 | 101972 |
| 74 | 8572 | 11.7 | 100292 | 6601 | 14.1 | 93074 |
| 75 | 7971 | 11.1 | 88478 | 6332 | 13.5 | 85482 |
| 76 | 8000 | 10.6 | 84800 | 6226 | 12.8 | 79693 |
| 77 | 7986 | 10.1 | 80659 | 6314 | 12.2 | 77031 |
| 78 | 7733 | 9.6 | 74237 | 6102 | 11.7 | 71393 |
| 79 | 7026 | 9.2 | 64639 | 5802 | 11.1 | 64402 |
| 80 | 7054 | 8.7 | 61370 | 5877 | 10.6 | 62296 |
| 81 | 6842 | 7.7 | 52683 | 5762 | 9.6 | 55315 |
| 82 | 6331 | 6.7 | 42418 | 5700 | 8.6 | 49020 |
| 83 | 5864 | 5.7 | 33425 | 5434 | 7.6 | 41298 |
| 84 | 5692 | 4.7 | 26752 | 5503 | 6.6 | 36320 |
| 85+ | 24676 | 1.7 | 43011 | 25461 | 3.3 | 84309 |
| Total | 353603 |  | 6404783 | 277973 |  | 5490903 |
| **Average number of years lost** |  |  | **18.1** |  |  | **19.8** |
